# Optimization of beef broth processing technology and isolation and identification of flavor peptides by consecutive chromatography and LC‐QTOF‐MS/MS

**DOI:** 10.1002/fsn3.1746

**Published:** 2020-06-26

**Authors:** Linhan Wang, Kaina Qiao, Yan Huang, Yuyu Zhang, Junfei Xiao, Wen Duan

**Affiliations:** ^1^ Beijing Advanced Innovation Center for Food Nutrition and Human Health Beijing Key Laboratory of Flavor Chemistry Beijing Laboratory for Food Quality and Safety Beijing Technology and Business University Beijing China; ^2^ School of International Studies Shandong Youth University of Political Science Jinan China

**Keywords:** beef broth, flavor peptide, liquid chromatography‐mass chromatograph, orthogonal optimization, sensory evaluation

## Abstract

To investigate the flavor peptides of beef broth obtained under optimized stewing conditions, separation procedures such as ultrafiltration, Sephadex G‐15 column chromatography, and reversed‐phase high‐performance liquid chromatography were employed to isolate the umami taste peptides. Sensory evaluation was combined with liquid chromatography–mass spectrometry to detect the flavor peptides. The optimization of the stewing process conditions was studied using the orthogonal method, which indicated that time had the most significant effect on the taste efficiency of sensory evaluation, followed by the mixed spices, sucrose, and salt. The optimized cooking conditions included 3.5 hr of cooking time, 1.800 g of sucrose, 2.125 g of salt, and 1.500 g of mixed spices. The results showed that six peptides, including SDEEVEH, AEVPEVH, GVDNPGHP, GSDGSVGPVGP, SDGSVGPVGP, and DEAGPSIVH, were detected in sample X1M1; and seven peptides, including VAPEEHPT, VVSNPVDIL, VGGNVDYK, PFGNTHN, EAGPSIVHR, VDFDDIQK, and DEAGPSIVH, were detected in sample X2M2. This study compared the flavor peptides in stewed beef before and after the optimization, and thus provided a basis for the improvement of beef processing technology.

## Graphical abstract

Sensory evaluation combined with liquid chromatograph quadrupole time‐of‐flight mass spectrometry/mass spectrometry to separate flavor peptides. The optimal stewed beef formula was obtained by sensory evaluation. The results showed that six peptides were detected in sample X1M1; and seven peptides were detected in sample X2M2.

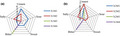

## 1 INTRODUCTION

Raw meat has a blood‐like flavor, due to the presence of blood salts and products of pyrolysis and saliva, with some overtones caused by the species, food, and environment of the animal (Dang, Gao, Ma, & Wu, 2015). The meat acceptance depends on all the senses of a consumer (Maughan, Tansawat, Cornforth, Ward, & Martini, 2012). Generally, taste and odor sensations are developed in the meat when it is exposed to heat and then masticated. Taste may be defined as a sensory attribute of the soluble substances that is perceptible via specific molecular receptors located on the tongue; they are the sweet, bitter, sour, salty, and umami sensations (Mungure, Bekhit, Birch, & Stewart, 2016; Narukawa et al., 2011). However, Aspartic acid (Asp, D), Glutamic acid (Glu, E), Proline (Pro, P), Alanine (Ala, A), Valine (Val, V), Methionine (Met, M), Arginine (Arg, R), and tartaric acid are the eight compounds that contribute more to the taste of stewed beef broth (Wang et al., 2020).

As a paradigmatic umami tastant, monosodium glutamate (MSG) seems to be slightly monotonous (umami taste only); however, umami peptides may exhibit multiple tastes or a variety of flavors besides the basic umami taste (Zhang, Venkitasamy, Pan, Liu, & Zhao, 2017; Zhuang et al., 2016). Certain umami peptides may also act as taste‐ or umami‐enhancing agents that are capable of intensifying sweetness, sourness, umaminess, and saltiness, including those derived from salts, glutamates, and acidulants (Schlichtherle‐Cerny & Amado, 2002; Zhuang et al., 2016). Taste‐active compounds have been identified by combining sensory evaluation with instrumental analysis. Such compounds include umami peptides in the enzymatic hydrolysate of deamidated wheat gluten (Schlichtherle‐Cerny & Amado, 2002; Zhang, Zhao, Su, & Lin, 2019), bitter products of the Maillard reaction (Schlichtherle‐Cerny & Amado, 2002), taste enhancer alapyridaine in beef broth (Ottinger & Hofmann, 2003), and kokumi peptides in yeast extract (Liu, Liu, He, Song, & Chen, 2015). Liu et al. (2015) reported that monitoring the heating temperature (> or <100°C) could enhance the meaty or broth‐like taste (i.e., umami and kokumi) of a Maillard reaction system that involved glucose and chicken peptides. Beksan et al. (2003) successfully isolated two compounds with an intense umami taste and excellent umami‐enhancing ability from a Maillard reaction system that involved glucose and L‐glutamic acid. Huang, Duan, Wang, Xiao, and Zhang (2019) identified five flavor peptides in stir‐fried beef: four octapeptides and one hexapeptide.

Umami peptides are naturally found in a wide variety of foods and have been proven to be essential for contributing to the taste of foods. The beef umami peptide (beefy meaty peptide, BMP) was detected from the gravy of papain‐treated beef meat in 1978, and it was confirmed by sensory evaluation that BMP enhances the taste of meat (Yamasaki & Maekawa, 1978, 2008). Two novel umami peptides, including an octapeptide and undecapeptide, were extracted from the peanut protein hydrolysate (Su et al., 2012). Further, Kang, Alim, and Song (2019) identified and characterized flavor peptides from beef enzymatic hydrolysates. They purified the enzymatic hydrolysates of beef using ultrafiltration (UF)/gel filtration chromatography (GFC)/reversed‐phase high‐performance liquid chromatography (RP‐HPLC) and identified 21 types of peptides. They selected six types of identified peptides for synthesis. Their results demonstrated that all six synthetic peptides that were reacted with xylose using the Maillard reaction exhibited a strong meaty delicious flavor and strong flavor enhancement ability. Beef broth is delicious and plays an important role in cooking. However, meat processing has a significant impact on the flavor of beef broth. To date, only a few studies have been reported on the flavor peptides present in the optimized beef broth.

In this study, the optimization of the process conditions of beef stewing was studied using an orthogonal method. The flavor peptides in stewed beef before and after the optimization were compared and analyzed. Along with sensory evaluation, separation procedures including UF, Sephadex G‐15 column chromatography, and RP‐HPLC were used to isolate the umami taste peptides. The molecular mass and amino acid sequences of the peptides were identified using liquid chromatograph quadrupole time‐of‐flight mass spectrometry/mass spectrometry (LC‐QTOF‐MS/MS).

## 2 MATERIALS AND METHODS

### 2.1 Materials and chemicals

The beef cuts (knuckle, with moisture, ash, protein, and fat contents of 70.8, 1.0, 21.9, and 1.1%, respectively and glucose, fructose, lactose, sucrose, and maltose contents of <0.1% each), Chinese prickly ash, green prickly ash, Cambodian cardamom, cumin, black pepper, white pepper, licorice, ginger, Chinese cassia, nutmeg, tangerine peel, greater galanga, fennel, small cardamom, amomum globosum loureiro, laurel, angelica dahurica, chili, dried hawthorn, tsao‐ko, and star anise were purchased from the Yonghui supermarket. The Welsh onion powder, onion powder, and coriander powder were obtained from the Shandong Fufeng Fermentation Co., Ltd. Salt and sucrose were purchased from the China National Salt Industry Group Co., Ltd. and Beijing Yulixing Commercial Trade Co., Ltd., respectively. Ultrapure water was obtained from the Wahaha. Acetonitrile (ACN, HPLC grade) and formic acid (analytical grade) were purchased from the Fisher Scientific and Sinopharm Chemical Reagent Co., Ltd, respectively. The Sephadex G‐15 was obtained from the Beijing RuiDaHengHui Science & Technology Development Co., Ltd. The HD‐A computer collector and HL‐2S constant‐current pump were obtained from Shanghai Huxi Co. Ltd.

### 2.2 Preparation of beef broth

The beef knuckle was cut into cubes of 3 cm and was added to an electric cooker (DGD32‐32BG; Tonze) with water; then, it was stewed using a nutrient soup model. The ratio of beef to water, cooking time, amount of salt and sucrose, and type and amount of spice were optimized using the single factor test and orthogonal optimization because they were the influencing factors of the umami taste of soup. After the completion of stewing, the beef broth was cooled to room temperature and then placed in a refrigerator maintained at 4°C for 24 hr. Subsequently, the surface oil was removed, and the beef broth samples were obtained.

Beef broth I (X1): 100.00 g of beef cuts and 150.00 g of water were added to an electric cooker and were stewed using a nutrient soup model for 3.5 hr. The treatment of the sample is described in “2.2. Preparation of beef broth.” This process was repeated 10 times. Ultimately, beef broth I (X1) sample was obtained.

Beef broth II (X2): 100.00, 1.800, 2.125, 1.500, and 150 g of beef cuts, sucrose, salt, mixed spices, and water, respectively, were added to an electric cooker and were stewed using a nutrient soup model for 3.5 hr. The treatment of the sample is described in “2.2. Preparation of beef broth.” This process was repeated 10 times. Ultimately, beef broth II (X2) sample was obtained.

### 2.3 Purification of flavor peptides from the beef broth using UF

**FIGURE 1:**
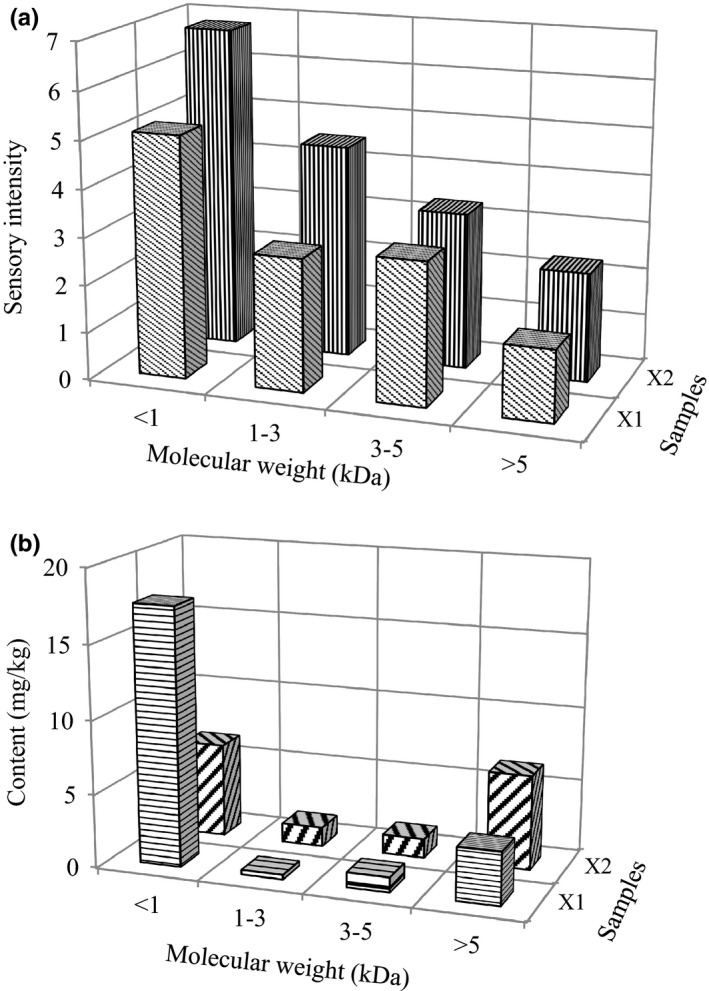
Sensory evaluation scores (a) and fraction content (b) of ultrafiltration fraction in stewed beef

Beef broths I and II were centrifuged (at below 4°C) in 50ml centrifuge tubes at 3,922 g (relative centrifugal force, RCF) for 15 min. The fat‐removed supernatant was then ultrafiltrated (at below 25°C, 0.2 MPa) using an UF device (Millipore). Each sample was divided into four fractions based on the molecular weight (>5, 3–5, 1–3, and <1 kDa). These fractions were then collected and lyophilized. The relative content of each UF component (mg/kg) was obtained using the ratio of the total weight of lyophilized powder to 1 kg of beef. After weighing, the samples were stored at room temperature for the sensory evaluation and subsequent separation and purification. Of the two samples, two ultrafiltrated fractions that have the strongest taste were selected using sensory evaluation and dissolved in ultrapure water, and solutions with a concentration of 20–150 mg/ml were prepared. The samples were then filtered through a 0.22‐μm‐nylon filter twice, and 1–2 ml of the samples was loaded on a Sephadex G‐15 gel filtration column (1.6 × 100 cm; Qingpu Huxi Instrument Factory) at a flow rate of 2 ml/min; here, ultrapure water was used as an eluent (25°C). The instrument model including the HD‐21‐2 UV detector and its operating conditions including UV absorption and sensitivity were referenced to the reports of Kong et al. (2017, 2019) and Strong, Osicka, and Comper (2005). As shown in Figure 1a, the four fractions of the two samples were collected separately, the sensory evaluation was carried out after freeze‐drying, and the fractions that have the strongest taste were selected for further separation by RP‐HPLC.

### 2.4 Purification of flavor peptides from the beef broth using RP‐HPLC

The most intense taste fraction obtained from beef broth samples I and II based on the results of sensory evaluation was further separated using LC3000 HPLC (Beijing Innovation Tongheng Technology Co., Ltd.) by employing a COSMOSIL 5C_18_‐MS‐Ⅱ column (10 (I.D.) × 250 mm, 5 μm; Nacalai Tesque) at 30°C to obtain several subfractions containing flavor peptides. The mobile phase consisted of mobile phase A (ACN) and mobile phase B (ultrapure water) (*V*_A_: *V*_B_ = 1:9), and it was eluted with an equal gradient at a flow rate of 1 ml/min. The injection volume was 1 ml, and the wavelength of the UV detector was 214 nm. In addition, each collected fraction was freeze‐dried.

### 2.5 Identification of peptides using LC‐MS/MS

The dried RP‐HPLC fractions were dissolved again in ultrapure water. Among these solutions, those with the most intense umami taste were subjected to LC‐MS analyses. The peptides were identified using nanoliquid chromatography‐tandem mass spectrometry by employing an Eksigent Nano‐LC 425 system (Eksigent of AB Sciex) and a quadrupole/time‐of‐flight (Q‐TOF) TripleTOF^®^ 5,600+ system (AB Sciex Instruments) equipped with a nano‐electrospray ionization (nESI) source.

The mobile phase consisted of mobile phase A (2% *V/V* ACN and 0.1% *V/V* formic acid in ultrapure water) and mobile phase B (2% *V/V* ultrapure water and 0.1% *V/V* formic acid in ACN). The elution gradients of mobile phase were 5%–18% B for 0–33 min, 18%–35% B for 33–46 min, 35%–80% B for 46–49 min, 80% B for 49–53 min, 95% B for 53–54 min, and 5% B for 54–60 min. First, 10 ml of the sample was injected into the nESI‐LC‐MS/MS system. This sample was preconcentrated on a ChromXP C18‐CL trap column (200 μm × 0.5 mm, 3 μm 120 Å; Eksigent of AB Sciex) at a flow rate of 0.3 μl/min. Subsequently, the trap column was automatically switched in‐line onto a nano‐HPLC capillary column (75 μm × 15 cm, 3 μm 120 Å, ChromXP C18‐CL; Eksigent of AB Sciex). Triplicates were run for each sample.

The column outlet was directly coupled to an nESI system. The Q‐TOF was operated in a positive polarity and an information‐dependent acquisition mode. A TOF‐MS scan with an accumulation time of 0.25 s and m/z of 50–1,500 was performed, following which 30 product ion scans with an accumulation time of 100 ms per MS/MS and m/z of 100–1,500 were performed. The dynamic exclusion time was 8 s for 30 min‐gradient and 12 s for 60 min‐gradient. The ion spray voltage was 2.4 kV, GS1 was 5 psi, curtain gas was at 30 psi, DP was 100, CES was 5, and the rolling CE was enabled.

### 2.6 Taste evaluation

Eleven panelists (six females and five males) aged between 21 and 25 years were selected from the Beijing Technology and Business University and were asked to rate the intensity of the taste qualities (sourness, sweetness, bitterness, saltiness, and umami) on a scale from 0 (not detectable) to 10 (strongly detectable). The concentration of the solution was according to the reports of Meyer, Dunkel, and Hofmann (2016) and Pu et al. (2020). The sensory evaluation was performed in a sensory panel room at 23 ± 2°C using the grading system (Han et al., 2017; Kang, Lee, & Park, 2014). The samples were analyzed using a score test that employs a 10‐point scale. The score indexes included the taste of sourness, sweetness, bitterness, saltiness, and umami. Thus, the sensory evaluation criteria were established. Taste solutions with different concentrations were numbered and arranged randomly. The panelists were required to accurately arrange the taste solutions with different concentrations in order from low to high. Based on the concentration of experimental sample, the taste score of the standard control solution was set at 5, including umami (3.50 mg/ml of salt and MSG, *m*_salt_: *m*_MSG_ = 1:1), sourness (0.025 mg/ml of citric acid), sweetness (1.00 mg/ml of sucrose), bitterness (0.001 mg/ml of quinine sulfate), and saltiness (3.50 mg/ml of salt).

Beef broth: The beef broth was heated, supplemented with 2% (*m*_beef_) salt, and placed at 40 ± 2°C for evaluation. The taste score of beef broth Ⅰ supplemented with 2% (*m*_beef_) salt was set at 5, including umami and overall tastes. The lyophilized UF and GFC fractions were dissolved and evaluated in ultrapure water at a concentration of 10 mg/ml. The scorecards, provided by the 11 judges, were used for the multivariate statistical analysis of all the descriptors at the end of each segment. Members of the sensory evaluation panel were asked to rinse their mouth with pure water and rest for more than 10 s between two samples.

## 3 RESULTS AND DISCUSSION

### 3.1 Optimization of single factor

The ratio of beef to water, cooking time, amount of salt and sucrose addition, and type and amount of spice were optimized individually using sensory evaluation by employing single factor experiments. The sensory evaluation results of beef broth obtained using the single factor test are depicted in Figure S1. With an increase in the added amount of sucrose, the score of umami and overall tastes increased until 1.750 g. When the added amount of sucrose was 2.000 g, the highest scores of umami and overall tastes were 7.25 and 7.50, respectively. The five high scores of umami and overall tastes were 7.00 and 7.07 (Chinese prickly ash), 7.03 and 7.11 (cumin), 6.98 and 7.09 (Welsh onion powder), 7.02 and 7.03 (onion powder), and 7.04 and 7.12 (coriander powder), respectively. Kranz, Viton, Smarrito‐Menozzi, and Hofmann (2018) added spices such as leeks, onions, celery, and cloves to make the traditional beef soup Pot‐au‐feu.

The sensory evaluation results of beef broth obtained using single factor test of five kinds of spices are depicted in Figure S2. As shown in Figures S1 and S2, the results of single factor optimization were as follows: the ratio of beef to water was 2:3 (*m*/*m*); the cooking time was 3.0 hr; the amount of salt and sucrose addition was 2.000 g; Chinese prickly ash addition was 0.200 g; the amounts of cumin, Welsh onion powder, onion powder, and coriander powder addition were 0.150, 0.300, 0.300, and 0.050 g, respectively.

### 3.2 Orthogonal optimization

The orthogonal experiment method was employed to select appropriate representative points from a large number of points to be tested, perform the experiments, and analyze the data based on an orthogonal table (Zhang, Xu, Li, Wen, & Yang, 2018). Based on the added amounts of five spices, an *L*_16_(4^5^) orthogonal array, which consisted of 16 rows and 5 columns, was chosen, and the results are listed in Table S1. Table S2 lists the corresponding variables and their values of sensory evaluation scores based on the orthogonal method. Table 1 shows the sensory evaluation scores based on the amounts of Chinese prickly ash, cumin, Welsh onion powder, onion powder, and coriander powder used in the orthogonal experiments. A maximum score of 6.37 and a minimum score of only 4.03 could be achieved. From this large difference in scores, it can be concluded that the amounts of spices can indeed significantly affect the sensory evaluation scores of beef broth.

**TABLE 1:** Results of the *L*_16_(4^5^) orthogonal experiment method on stewing beef

Run order	Chinese prickly ash (g)	welsh onion (g)	Cumin (g)	Onion (g)	Coriander (g)	Scores of sensory evaluation
1	0.200	0.200	0.145	0.350	0.045	5.51
2	0.200	0.250	0.150	0.400	0.050	6.23
3	0.200	0.300	0.155	0.450	0.055	5.52
4	0.200	0.350	0.160	0.500	0.060	5.47
5	0.250	0.200	0.150	0.450	0.060	5.56
6	0.250	0.250	0.145	0.500	0.055	4.03
7	0.250	0.300	0.160	0.350	0.050	6.07
8	0.250	0.350	0.155	0.400	0.045	6.26
9	0.300	0.200	0.155	0.500	0.050	4.67
10	0.300	0.250	0.160	0.450	0.045	5.20
11	0.300	0.300	0.145	0.400	0.060	4.71
12	0.300	0.350	0.150	0.350	0.055	5.77
13	0.350	0.200	0.160	0.400	0.055	5.39
14	0.350	0.250	0.155	0.350	0.060	4.49
15	0.350	0.300	0.150	0.500	0.060	5.84
16	0.350	0.350	0.145	0.450	0.050	6.37
K_1_	5.682	5.282	5.155	5.460	5.702	
K_2_	5.480	4.988	5.850	5.647	5.835	
K_3_	5.088	5.535	5.235	5.662	5.178	
K_4_	5.522	5.968	5.532	5.002	5.057	
*R*	0.594	0.980	0.695	0.660	0.778	
Rank	R5	R1	R3	R4	R2	6.82

Range analysis of the response data was performed to measure the optimal values for these variables. The means of sensory evaluation scores (*K_i_*) for the 4 levels (*I* = 1, 2, 3, 4) of each factor are listed in Table 1. For each factor, larger mean value indicates that the particular level contributes more to the sensory evaluation scores (Zhang et al., 2018). The highest scores of sensory evaluation were obtained for the following factors: 0.200 g of Chinese prickly ash, 0.350 g of Welsh onion powder, 0.150 g of cumin, 0.450 g of onion powder, and 0.050 g coriander powder. From Table 1, it is evident that the amount of Welsh onion powder has the most significant effect on the sensory evaluation scores, followed by the amounts of coriander powder, cumin, onion powder, and Chinese prickly ash.

Figure S3a shows the main effects plot for the mean values of each factor. It was observed that the Welsh onion powder has the most significant effect on the sensory evaluation scores; this is in accordance with the range analysis results. Thus, the ratio of spices was *m*_welsh onion_: *m*_coriander_: *m*_cumin_: *m*_onion_: *m*_Chinese prickly ash_ = 4:7:3:9:1. Based on the cooking condition parameters, an *L*_9_(3^4^) orthogonal array was chosen, and the results are listed in Table S3. The optimal cooking conditions were obtained from the maximum possible score of sensory evaluation (Zhang et al., 2018).

Table S4 lists the corresponding variables and their values of sensory evaluation scores based on the orthogonal method. Table 2 shows the sensory evaluation scores based on the cooking time and amounts of sucrose, salt, and mixed spices obtained from the orthogonal experiments. The highest scores of sensory evaluation were obtained for the following factors: 3.5 hr of cooking time, 1.800 g of sucrose, 1.875 g of salt, and 1.500 g of mixed spices. Generally, the cooking time has the most significant effect on the sensory evaluation scores, followed by mixed spices, sucrose, and salt (Zhang et al., 2018). Figure S3b shows the main effects plot for the mean values of each factor. In Figure S3b, the relative slope of the cooking time profiles is larger, further indicating that the cooking time has the most significant effect on the sensory evaluation scores; this is in accordance with the range analysis results. Thus, the optimized cooking conditions are as follows: 3.5 hr of cooking time, 1.800 g of sucrose, 2.125 g of salt, and 1.500 g of mixed spices.

**TABLE 2:** The cooking condition and sensory evaluation results of stewing beef

Run order	Time (hr)	Sucrose (g)	Salt (g)	Mixed spice (g)	Scores of sensory evaluation
1	2.5	1.750	1.875	1.450	7.41
2	2.5	1.750	2.000	1.450	7.56
3	2.5	1.800	2.125	1.550	7.54
4	3.0	1.800	2.000	1.550	7.25
5	3.0	1.700	2.125	1.450	7.26
6	3.0	1.750	1.875	1.500	7.82
7	3.5	1.750	2.125	1.500	7.99
8	3.5	1.700	1.875	1.550	7.75
9	3.5	1.800	2.000	1.450	7.93
K1	7.503	7.550	7.660	7.533	
K2	7.443	7.523	7.580	7.790	
K3	7.890	7.763	7.597	7.513	
*R*	0.447	0.240	0.080	0.277	
Rank	R1	R3	R4	R2	8.24

### 3.3 Fractionation using UF

Several flavor peptides are generated in a raw beef meat and stewed beef as a consequence of muscle protein degradation. Dang et al. (2015) obtained umami taste peptides from the fractions (with *M*_W_ < 5 kDa) of ham. To study the umami effect of peptides, beef broths I and II were divided into four peptide fractions using UF membranes based on the *M*_W_ range (>5, 3–5, 1–3, and <1 kDa, respectively) (Figure 1). All the recovered fractions were lyophilized and dissolved again for sensory evaluation. The sensory evaluation of the four peptide fractions was then performed based on their umami taste. As shown in Figure 1a, the fractions with *M*_W_ < 1 kDa exhibit the highest sensory evaluation scores; that is, 5.13 for X1 and 6.80 for X2. It was observed that with the increasing *M*_W_, the sensory evaluation scores decreased. The fractions with *M*_W_ < 1 and 1–3 kDa contributed to the umami taste (Su et al., 2012). The observation that the fraction with *M*_W_ < 1 kDa has the most umami taste is consistent with the previous studies (Dang et al., 2015). As shown in Figure 1b, the fractions with *M*_W_ < 1 kDa exhibit the highest contents; that is, 17.52 g/kg for X1 and 32.01 g/kg for X2.

### 3.4 Fractionation using GFC

**FIGURE 2:**
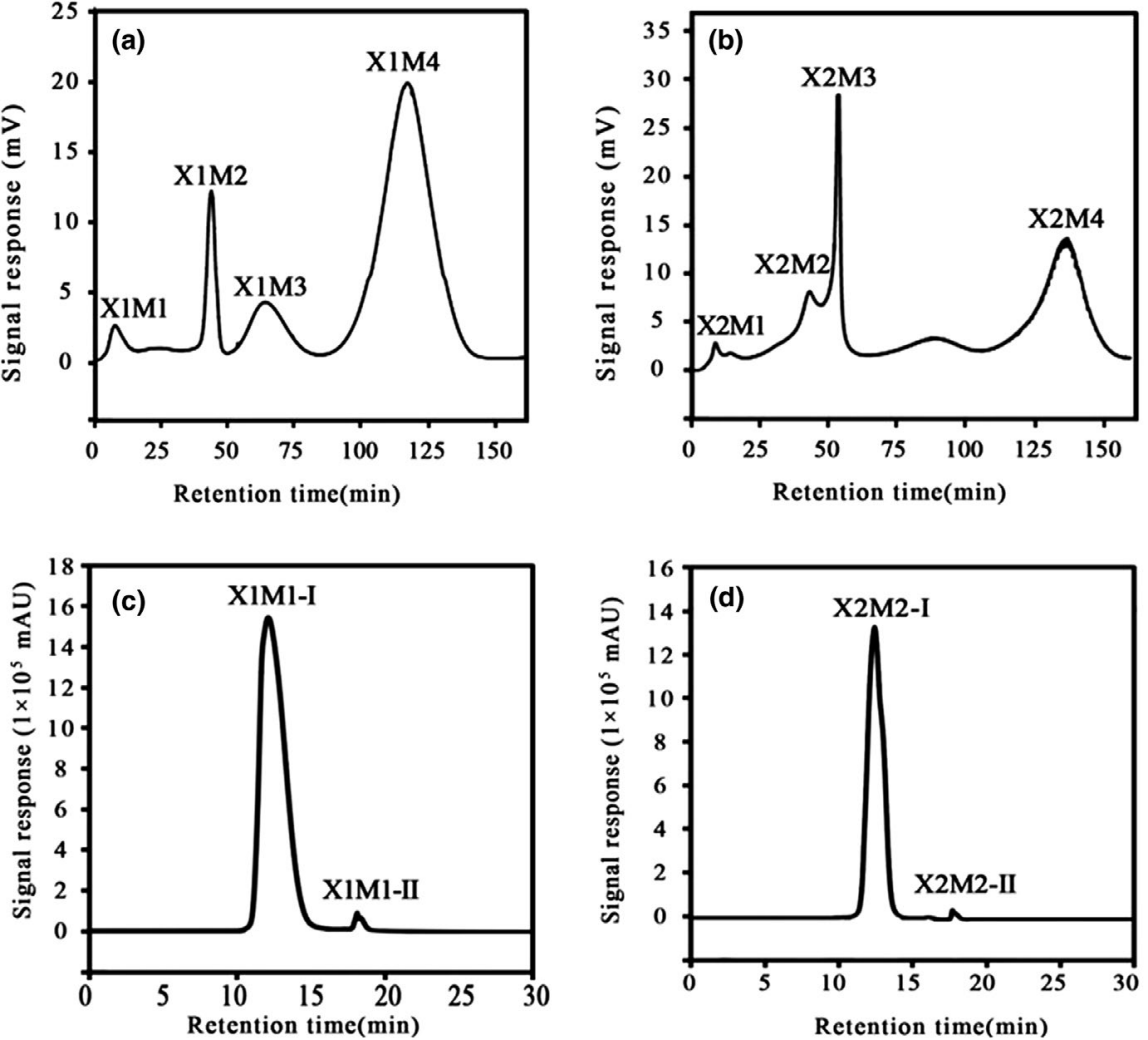
Purification of crude peptide with <1 kDa in beef broth by Sephadex G‐15 and reversed‐phase high‐performance liquid chromatography (RP‐HPLC). (a) Sephadex G‐15 gel filtration chromatogram of ultrafiltration (UF) fraction with molecular weight (*M*_W_) <1 kDa obtained from stewed beef blank experiment; (b) Sephadex G‐15 gel filtration chromatogram of UF fraction with *M*_W_ < 1 kDa obtained from stewed beef orthogonal optimal experiment; (c) RP‐HPLC chromatogram of gel filtration fraction with the highest umami score obtained from stewed beef soup blank experiment; (d) RP‐HPLC chromatogram of gel filtration fraction with the highest umami score obtained from stewed beef soup orthogonal optimal experiment

The most intense umami peptide fractions (with *M*_W_ < 1 kDa) were further separated using the Sephadex G‐15 column chromatography (Figure 2a,b). The fraction (with *M*_W_ < 1 kDa) of X1 was further separated into four subfractions (X1M1, X1M2, X1M3, and X1M4) (Figure 2a). The fraction (with *M*_W_ < 1 kDa) of X2 was further separated into four subfractions (X2M1, X2M2, X2M3, and X2M4) (Figure 2b).

**FIGURE 3:**
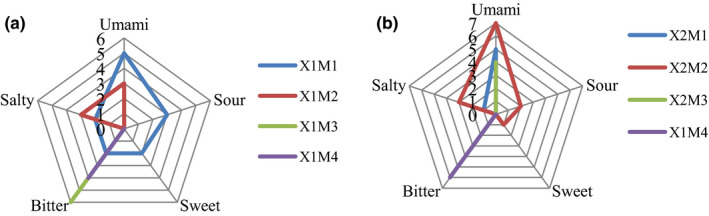
Sensory evaluation scores of Sephadex G‐15 fraction in beef broth. (a) Sensory evaluation radar chart of ultrafiltration (UF) separation components from stewed beef blank experiment; (b) sensory evaluation radar chart of UF separation components from stewed beef orthogonal optimal experiment

The sensory evaluation scores of four subfractions of beef broth are displayed in Figure 3. As shown in Figure 3a, the total taste score of X1M1 is 14, which is much higher than those of the other subfractions of X1. Subfraction X1M1 exhibited umami, sweet, sour, and bitter tastes; among these, the umami taste was dominant. Subfraction X1M2 exhibited a slightly umami and salty taste. Subfractions X1M3 and X1M4 exhibited mainly a bitter taste, and X1M3 was more bitter than X1M4. Therefore, X1M1 was selected for the next step of purification using RP‐HPLC.

As shown in Figure 3b, the total taste score of X2M2 is 13, which is much higher than those of the other subfractions of X2. Subfraction X2M2 exhibited umami, sweet, sour, and salty tastes; among these, the umami taste was dominant. Subfractions X2M1 and X2M3 exhibited a umami taste; however, its intensity was lower than that of X2M2. Subfraction X2M4 exhibited an extremely strong bitter taste. Therefore, X2M2 was selected for the next step of purification using RP‐HPLC.

### 3.5 Purification of taste peptides using RP‐HPLC

To identify the umami taste effect, subfractions X1M1 and X2M2 were further fractionated using a RP‐HPLC system including a preparative C18 column. As shown in Figure 2c, peak of X1M1 is separated into two peaks; that is, those of X1M1‐Ⅰ and X1M1‐Ⅱ. As shown in Figure 2d, peak of X2M2 is separated into two peaks; that is, those of X2M2‐Ⅰ and X2M2‐Ⅱ. Subfractions X1M1‐Ⅰ, X1M1‐Ⅱ, X2M2‐Ⅰ, and X2M2‐Ⅱ were collected and lyophilized for LC‐MS identification.

### 3.6 Identification of taste peptides using LC‐QTOF‐MS/MS

The molecular mass and sequence of the peptides of four subfractions (X1M1‐Ⅰ, X1M1‐Ⅱ, X2M2‐Ⅰ, and X2M2‐Ⅱ) were identified using LC‐QTOF‐MS/MS and are shown in Table 3. The MS/MS spectra of the purified peptides of all four subfractions are shown in Figure S4. The principle of LC‐QTOF‐MS/MS is that the high‐energy collision‐induced dissociation of peptides generates b‐type and y‐type ions when peptides are cleaved at an amide bond (Zhuang et al., 2016). ProteinPilot software was used to retrieve the collected data, and the secondary spectrum was matched with the sequence in the Bovine (UP000009136) using the search results. On account of repeated three times on isolation and purification employed to generate X1M1‐Ⅰ, X1M1‐Ⅱ, X2M2‐Ⅰ, and X2M2‐Ⅱ in this experiment, the matrix interference was reduced significantly; this can increase the feasibility and accuracy of obtaining a reliable sequence matching and structural characterization of peptides.

**TABLE 3:** The sequence of peptide in beef broth

Samples	No.	Sequence	Conf	*M*_W_
X1M1‐I	1	SDEEVEH	96.2	885.3352
	2	AEVPEVH	88.9	779.3813
	3	GVDNPGHP	77	791.3582
X1M1‐II	1	GSDGSVGPVGP	99	927.4297
	2	AEVPEVH	88.9	779.3813
	3	DEAGPSIVH	82.2	923.4349
	4	SDGSVGPVGP	69.8	870.4083
X2M2‐I	1	VAPEEHPT	97.7	878.4134
	2	VVSNPVDIL	87.3	954.5386
X2M2‐II	1	VGGNVDYK	99	850.4185
	2	PFGNTHN	99	785.3456
	3	EAGPSIVHR	99	964.509
	4	VDFDDIQK	99	978.4658
	5	DEAGPSIVH	98.9	923.4349

Note

“Conf,” The degree of match between detected ionic fragments and sequences in Bovine (UP000009136).

The abbreviation of amino acid: A, Ala, Alanine; C, Cys, Cysteine; D, Asp, Aspartic acid; E, Glu, Glutamic acid; F, Phe, Phenylalanine; G, Gly, Glycine; H, His, Histidine; I, Ile, Isoleucine; K, Lys, Lysine; L, Leu, Leucine; M, Met, Methionine; N, Asn, Asparagine; P, Pro, Proline; Q, Gln, Glutamine; R, Arg, Arginine; S, Ser, Serine; T, Thr, Threonine; V, Val, Valine; W, Trp, Tryptophan; Y, Tyr, Tyrosine.

As shown in Table 3, three peptides are identified in X1M1‐Ⅰ, four in X1M1‐Ⅱ, two in X2M2‐Ⅰ, and five in X2M2‐Ⅱ. The polybasic sodium salt formed by the combination of Glu (E), Glutamine (Gln, Q), Asp (D), and Asparagine (Asn, N) or combined with Threonine (Thr, T), Serine (Ser, S), Met (M), Glycine (Gly, G), and Ala (A) are umami; for example, Glu‐Glu (EE), Glu‐Ser (ES), Glu‐Asp (ED), Glu‐Thr (ET), Ser‐Glu‐Gln (SEQ), and Glu‐Gln‐Gln (EQQ) (Sentandreu et al., 2003). Ohyama, Ishibashi, Tamura, Nishizaki, and Okai (1988) synthesized the peptide of AEA and discovered the umami taste. Nakata et al. (1995) studied the role of basic Lysine (Lys, K)‐Gly (KG) and acidic Asp‐Glu‐Glu (DEE) fragments in delicious peptides. The results demonstrated that these two fragments play an important role in the taste production and intensity of delicious peptides. The localization of the cations of basic fragments and the anions of acidic fragments can produce umami or salty tastes. Five delicious peptide analogs, including SLAKGDEE, SLADEEKG, KGLAEE, KGDEE, and EEDGK, have been synthesized, and they are known to have umami and/or salty taste. In this study, we identified the following peptide sequences: SDEEVE in X1M1‐Ⅰ, DEAGPSIVH in X1M1‐Ⅱ, VAPEEHPT in X2M2‐Ⅰ, and DEAGPSIVH in X2M2‐Ⅱ, and it was observed that all of them have umami structures: EE or DEE.

It has been observed that most peptides containing hydrophobic amino acids, including Phenylalanine (Phe, F), Tyrosine (Tyr, Y), Leucine (Leu, L), Val, Pro, Ala, Tryptophan (Trp, W), Gly, Met, and Isoleucine (Ile, I), release a bitter taste (Sentandreu et al., 2003). Three peptides of X1M1‐I and X1M1‐II had a Pro terminal, and Pro was found in all the peptides of X2M2‐I and three peptides of X2M2‐II. All the peptides of X2M2‐II contained a larger number of hydrophobic amino acids, including Phe, Tyr, Ala, and Ile, and almost all of the identified taste peptides had Val. According to the related research, some of these twelve identified peptides were already detected in other studies. AM and VE had already been identified in Spanish dry‐cured ham, and they exhibited bitter and sour tastes, respectively (Sentandreu et al., 2003).

## 4 CONCLUSIONS

The optimum beef‐stewing conditions were obtained using a single factor test and an orthogonal experiment. These conditions included 100 g of knuckle meat, 150 g of water, 1.80 g of sucrose, 2.125 g of salt, 1.50 g of spices (*m*_welsh onion_: *m*_coriander_: *m*_cumin_: *m*_onion_: *m*_Chinese prickly ash_ = 4:7:3:9:1), stewing temperature of 100°C, and stewing time of 3.5 hr. Taste peptides of stewed beef blank group beef broth I (X1) and stewed beef orthogonal optimal group beef broth II (X2) were isolated, purified, and identified using UF, Sephadex G‐15 column chromatography, RP‐HPLC, and LC‐Q‐TOF‐MS/MS. By combining the sensory evaluation with LC‐MS, six peptides, including SDEEVEH, AEVPEVH, GVDNPGHP, GSDGSVGPVGP, SDGSVGPVGP, and DEAGPSIVH, were detected in sample X1M1; and seven peptides, including VAPEEHPT, VVSNPVDIL, VGGNVDYK, PFGNTHN, EAGPSIVHR, VDFDDIQK, and DEAGPSIVH, were identified in sample X2M2. The presence of some of these peptides with specific taste in the beef broth soluble fraction indicates that they may contribute significantly to taste or even interact with volatile compounds, affecting the entire flavor.

## CONFLICT OF INTEREST

The authors declare that they have no conflict of interest.

## ETHICAL APPROVAL

This study does not involve any human or animal testing.

## Supporting information

Supplementary Material
