# Supplementary material for: Optimization of beef broth processing technology and isolation and identification of flavor peptides by consecutive chromatography and LC‐QTOF‐MS/MS
Source: Food Sci Nutr. 2020 Jun 26;8(8):4463–71. doi: 10.1002/fsn3.1746 (PMC7455977; doi:10.1002/fsn3.1746)
Supplement: Supplementary file 1 — Supplementary Material [file FSN3-8-4463-s001.docx]

**Supplement materials**

**Figures**

|  |  |
| --- | --- |
|  |  |
|  | |

**Fig.S1** Sensory evaluation results of stewing beef by different single factor test. (a: beef cuts to water ratio; b: cooking time; c: the amount of salt addition; d: the amount of sucrose addition; e: the amount of spices addition) (1. Chinese prickly ash, 2. green prickleyash, 3. cambodian cardamom, 4. cumin, 5. black pepper, 6. white pepper, 7. Licorice, 8. Ginger, 9. Chinese cassia, 10. welsh onion, 11. nutmeg, 12. Onion, 13. Coriander, 14. Tangerine peel, 15. greater galanga, 16. fennel, 17. small cardamon, 18. amomum globosum loureiro, 19. Laurel, 20. angelica dahurica, 21. chilli, 22. dried hawthorn, 23. tsao-ko, 24. star anise; Different letters between columns represent significant differences between cultivars (*P* <0.05).)

|  |  |
| --- | --- |
|  |  |
|  | |

**Fig.S2** Sensory evaluation results of beef broth by single factor test of 5 kind spices. (a: Chinese prickly ash; b: cumin; c: welsh onion; d: onion; e: coriander; Different letters between columns represent significant differences between cultivars (*P* <0.05).)

**
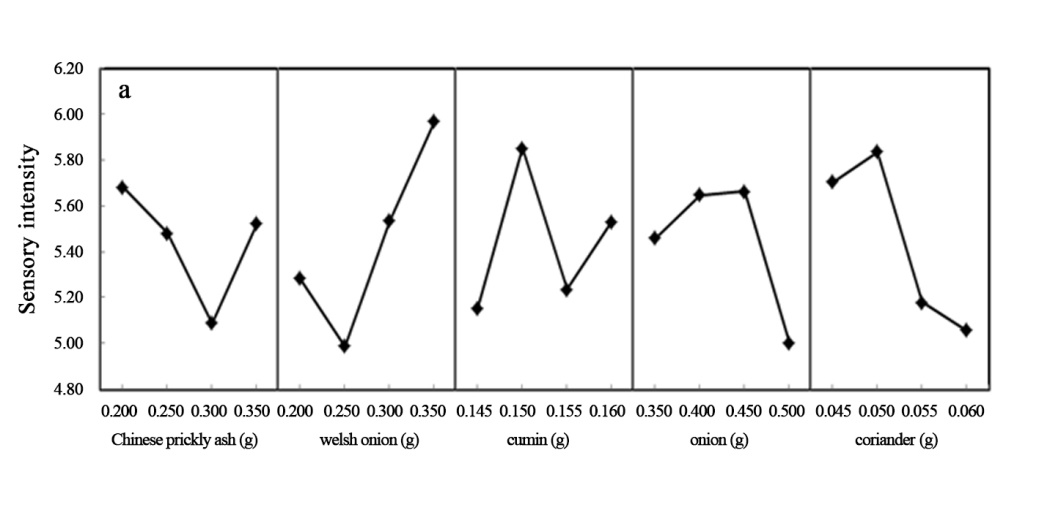
**

**
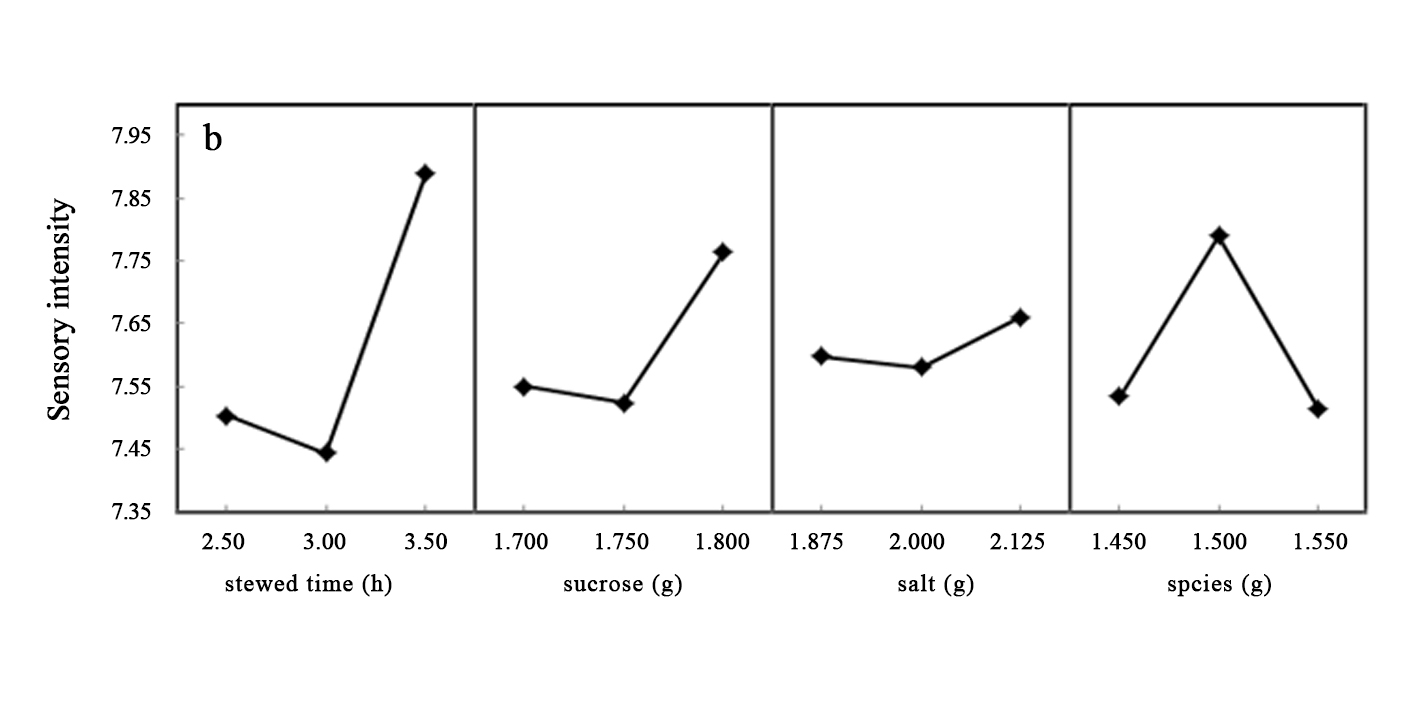
Fig.S3** Response graph for the mean values of each factor. (a: *L*_16_(4^5^); b: *L*_9_(3^4^)).

| **a** | **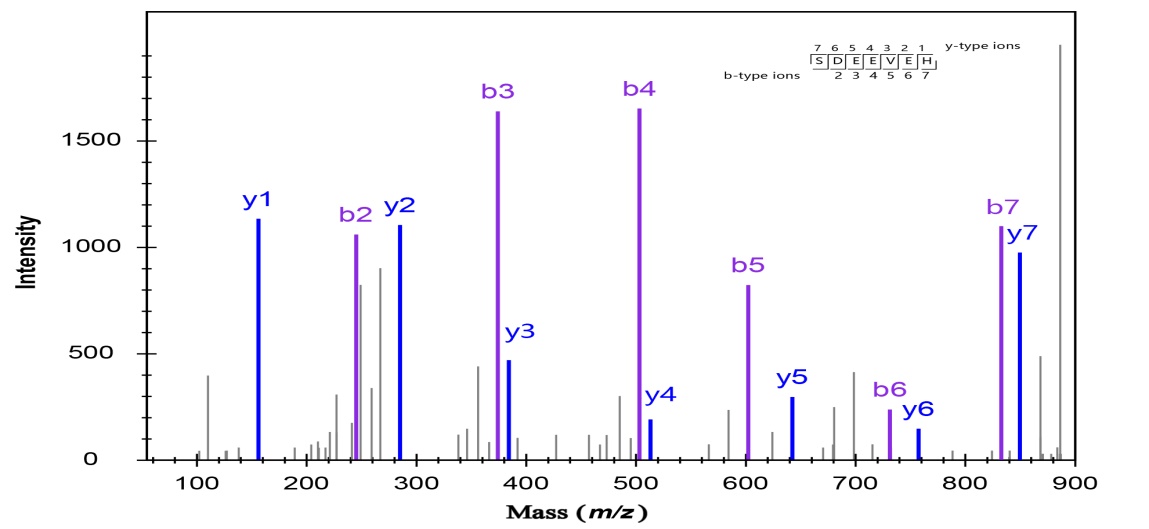** |
| --- | --- |
| **b** | **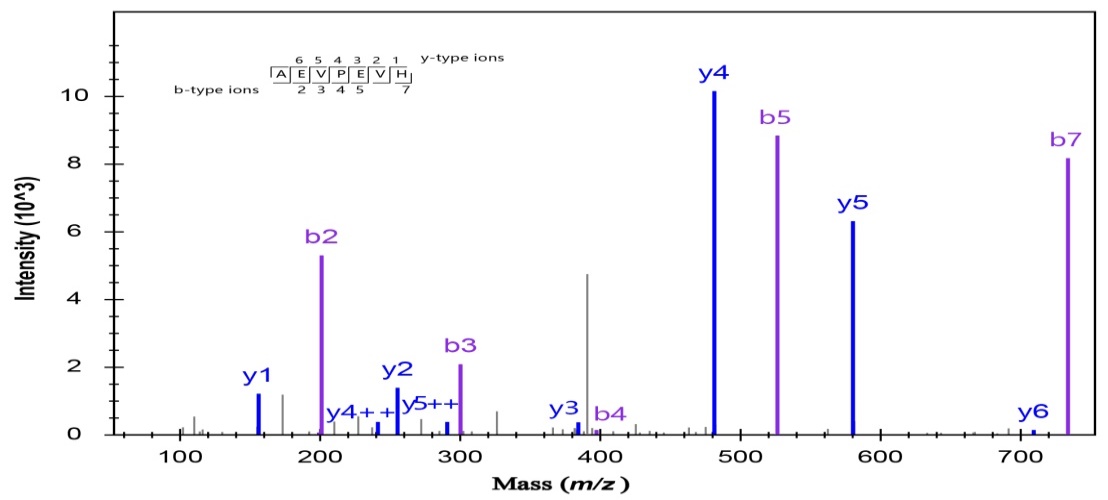** |
| **c** | **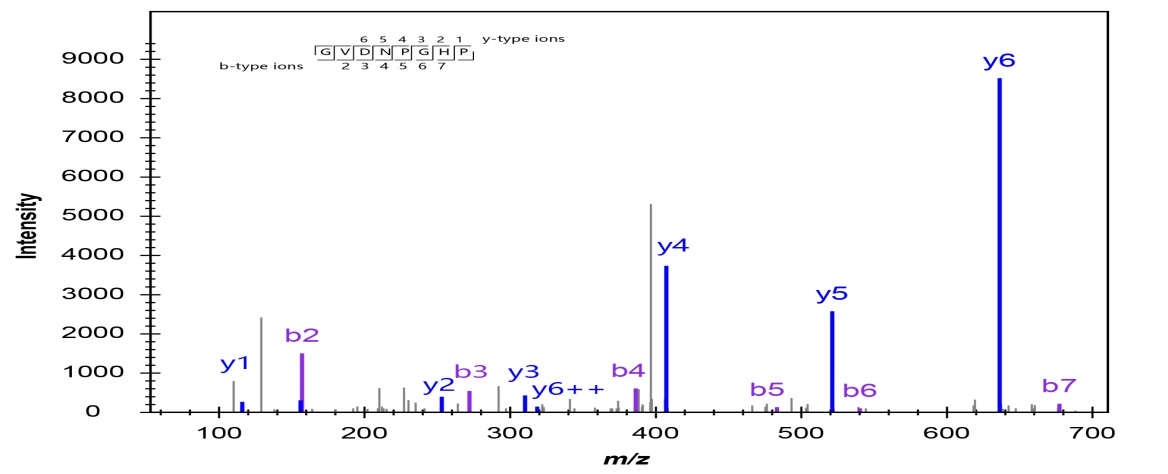** |
| **d** | **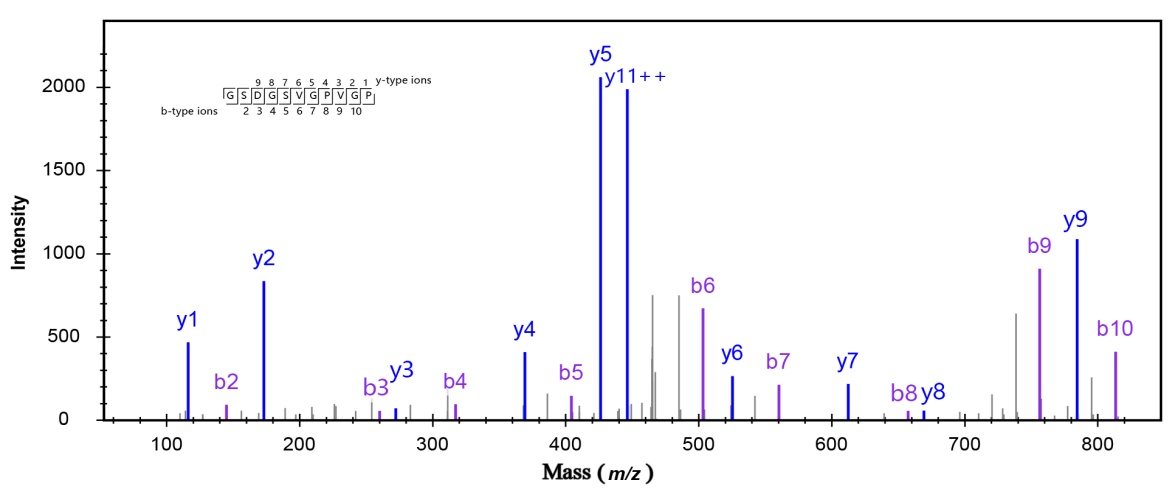** |
| **e** | **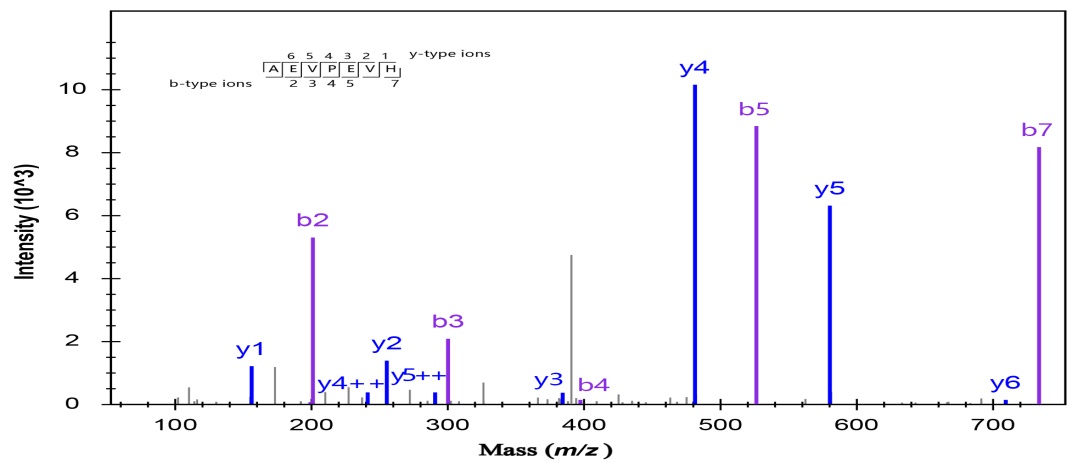** |
| **f** | **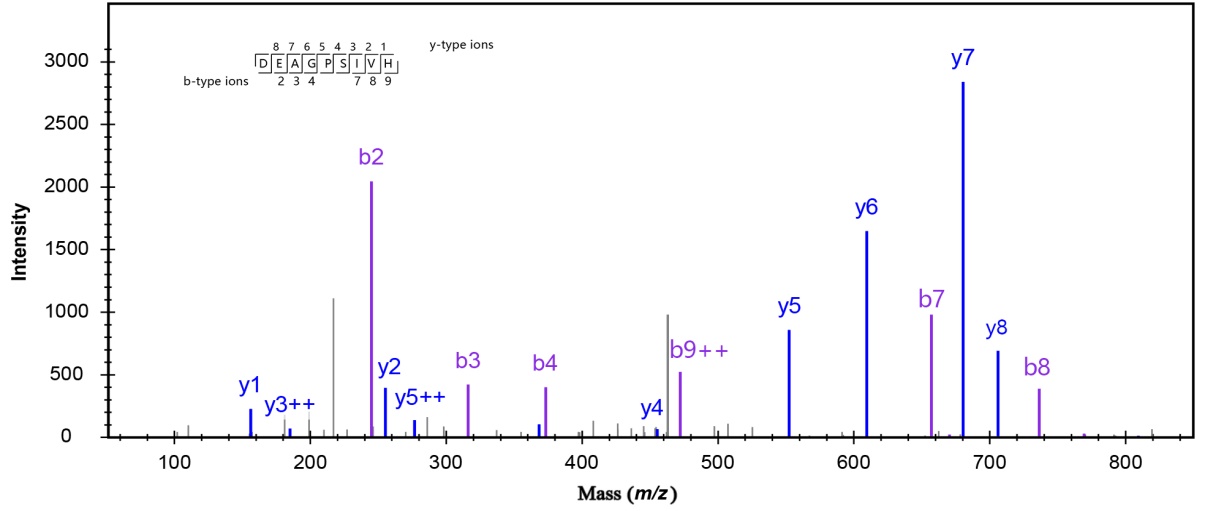** |
| **g** | **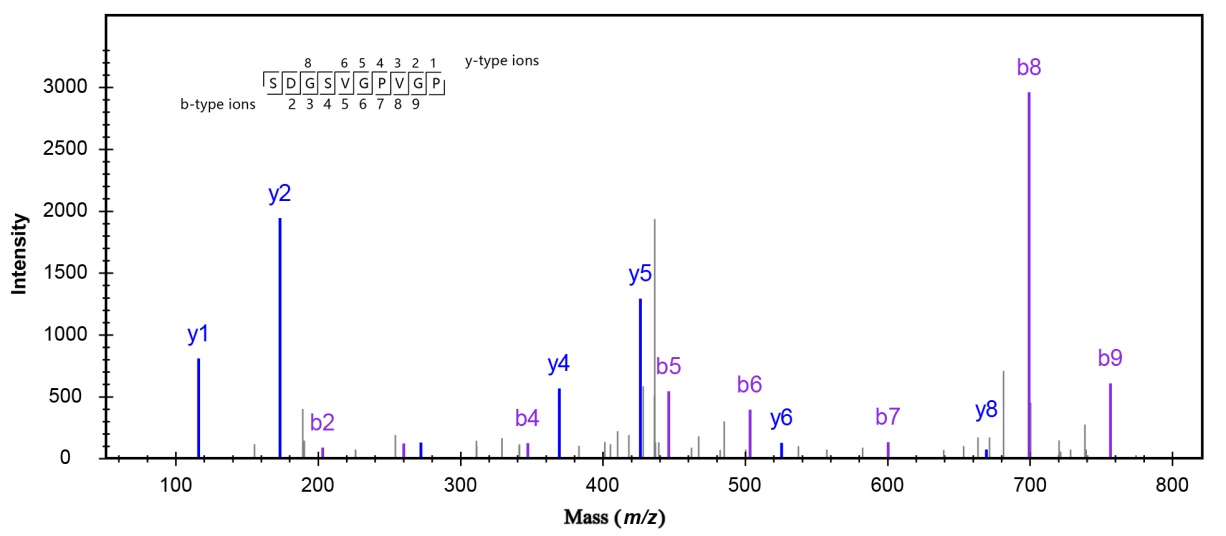** |
| **h** | 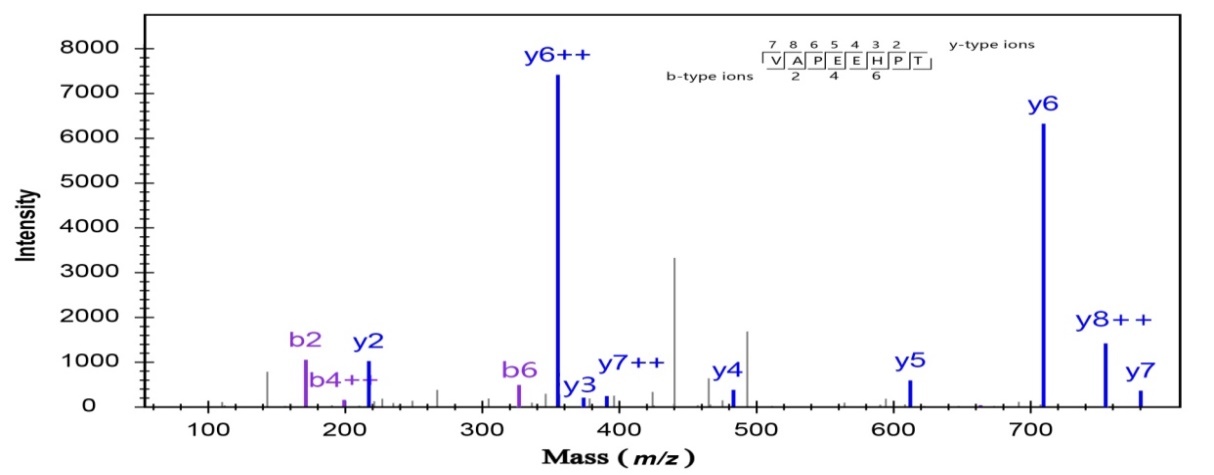 |

| **i** | 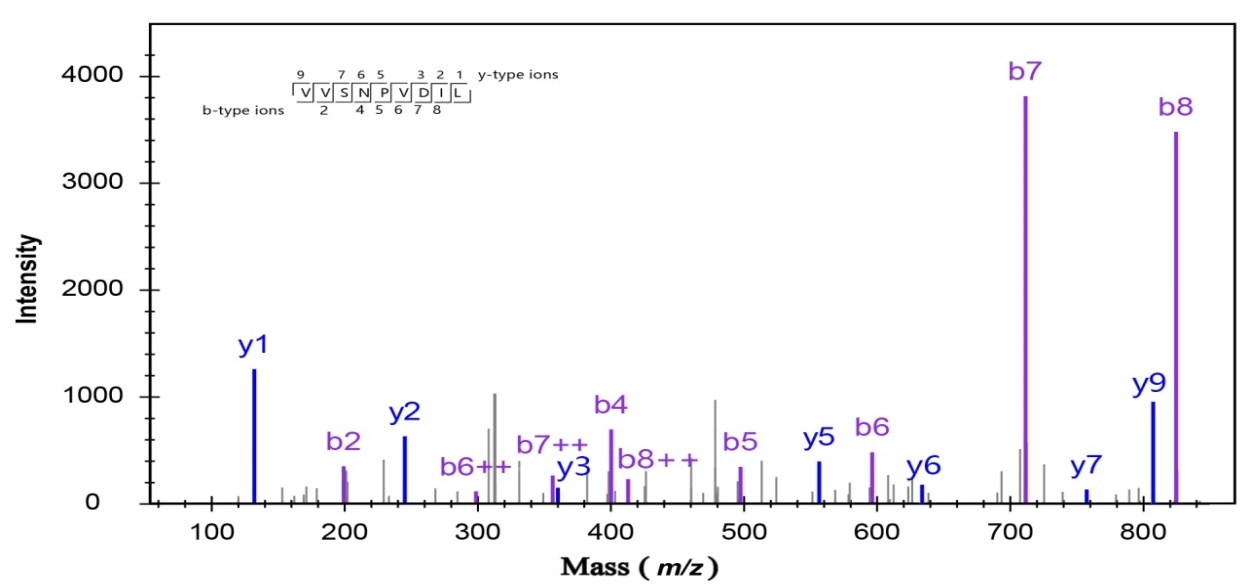 |
| --- | --- |
| **j** | 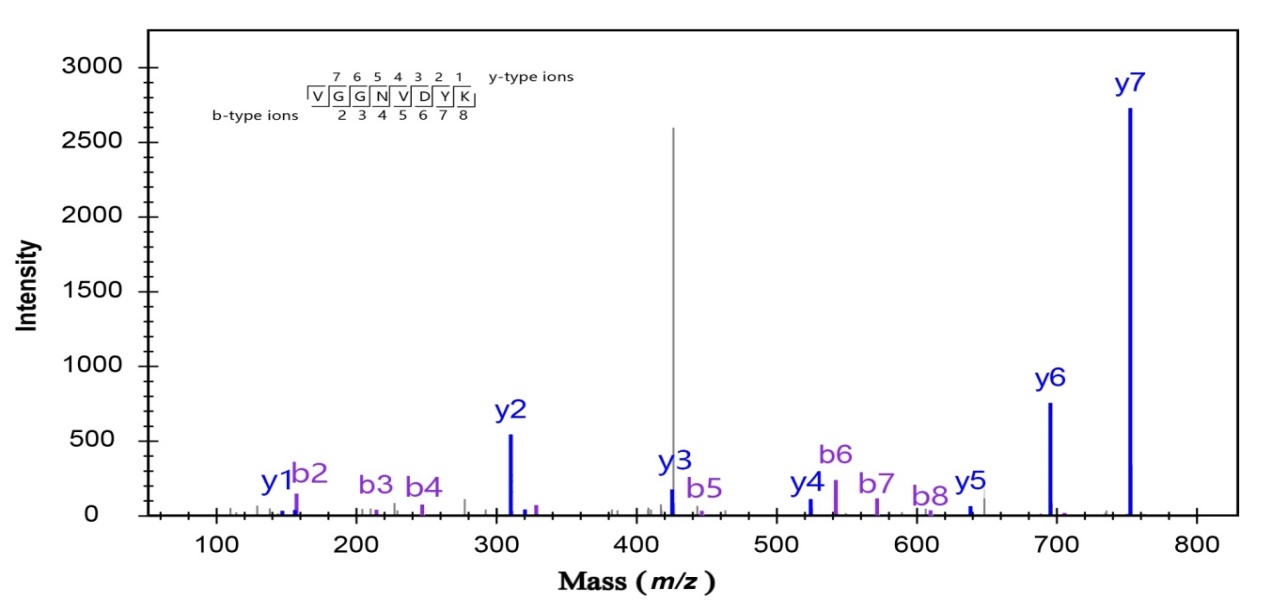 |
| **k** | 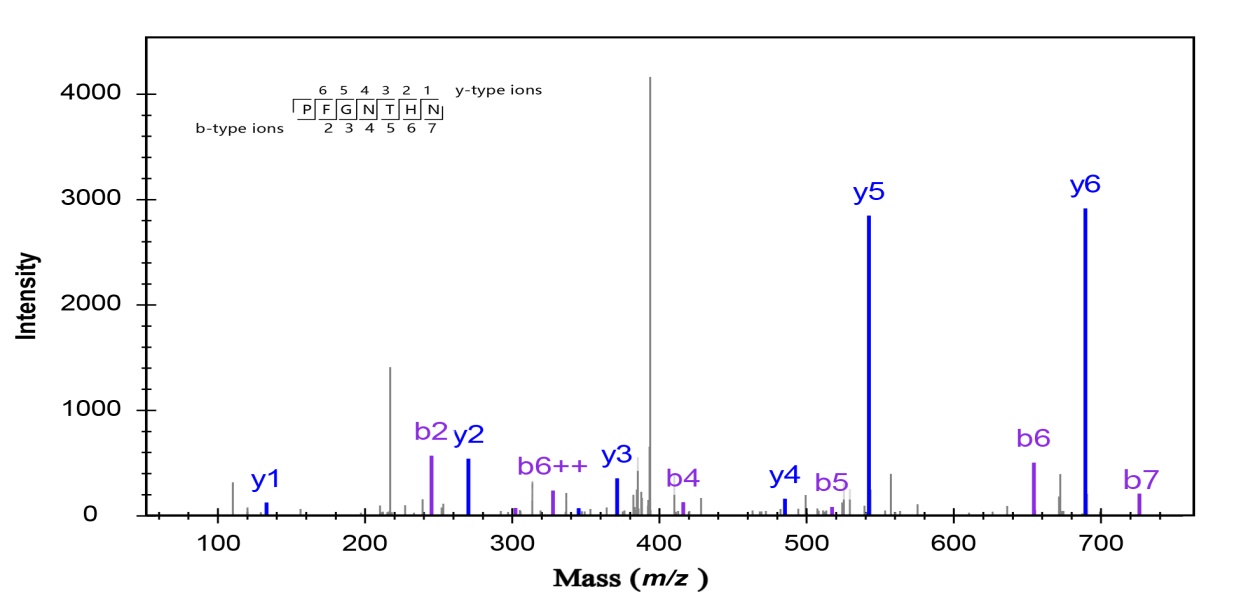 |

| **l** | 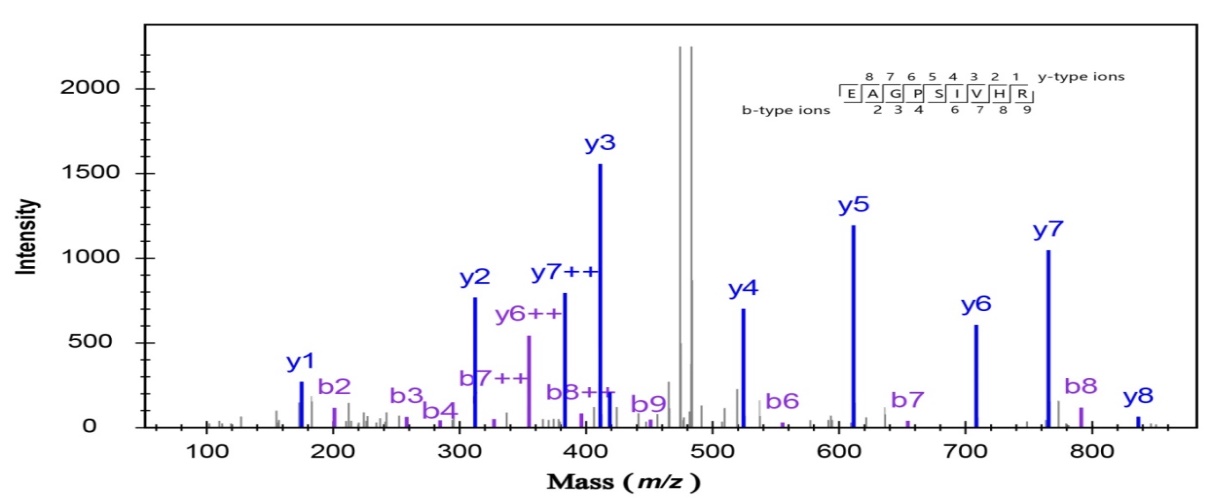 |
| --- | --- |
| **m** | 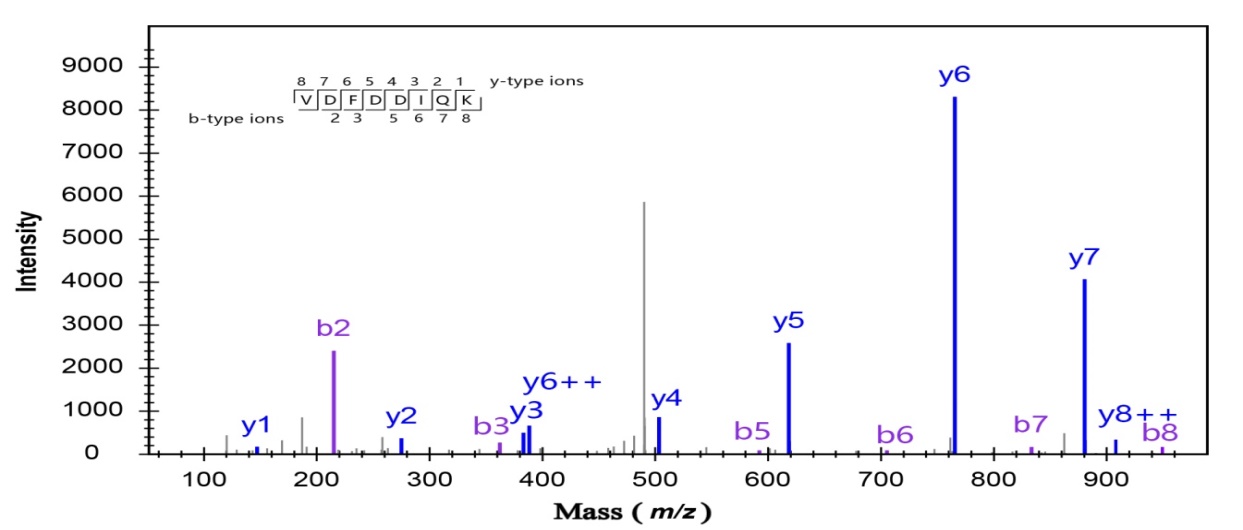 |
| **n** | 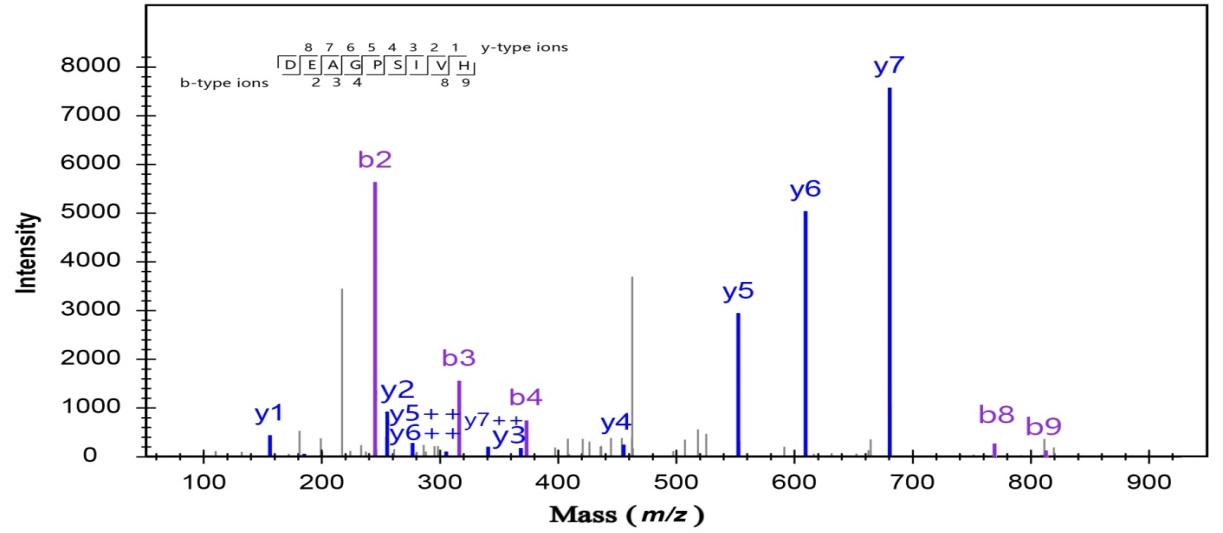 |

**Fig. S4** MS/MS spectra of purified peptides (a-c: the peptides from X1M1-Ⅰ, d-g: the peptides from X1M1-Ⅱ, h-i: the peptides from X2M2-Ⅰ, j-n: the peptides from X2M2-Ⅱ; The b and y represented the ions generated from peptides.).

**Tables**

**Table S1** *L*_16_(4^5^) orthogonal array of the orthogonal experiment method.

| Experiment order | Parameter level | | | | |
| --- | --- | --- | --- | --- | --- |
|  | (A) | (B) | (C) | (D) | (E) |
| 1 | 1 | 1 | 1 | 1 | 1 |
| 2 | 1 | 2 | 2 | 2 | 2 |
| 3 | 1 | 3 | 3 | 3 | 3 |
| 4 | 1 | 4 | 4 | 4 | 4 |
| 5 | 2 | 1 | 2 | 3 | 4 |
| 6 | 2 | 2 | 1 | 4 | 3 |
| 7 | 2 | 3 | 4 | 1 | 2 |
| 8 | 2 | 4 | 3 | 2 | 1 |
| 9 | 3 | 1 | 3 | 4 | 2 |
| 10 | 3 | 2 | 4 | 3 | 1 |
| 11 | 3 | 3 | 1 | 2 | 4 |
| 12 | 3 | 4 | 2 | 1 | 3 |
| 13 | 4 | 1 | 4 | 2 | 3 |
| 14 | 4 | 2 | 3 | 1 | 4 |
| 15 | 4 | 3 | 2 | 4 | 1 |
| 16 | 4 | 4 | 1 | 3 | 2 |

**Table S2** Parameters and levels of the *L*_16_(4^5^) orthogonal experiment method.

| Parameters | Level 1 | Level 2 | Level 3 | Level 4 |
| --- | --- | --- | --- | --- |
| (A) Chinese prickly ash powder (g) | 0.200 | 0.250 | 0.300 | 0.350 |
| (B) welsh onion powder (g) | 0.200 | 0.250 | 0.300 | 0.350 |
| (C)cumin powder (g) | 0.145 | 0.150 | 0.155 | 0.160 |
| (D) onion powder (g) | 0.350 | 0.400 | 0.450 | 0.500 |
| (E) coriander powder (g) | 0.045 | 0.050 | 0.055 | 0.060 |

**Table S3** *L*_9_(3^4^) orthogonal array of the orthogonal experiment method.

| Experiment order | Parameter level | | | |
| --- | --- | --- | --- | --- |
|  | (E) | (F) | (G) | (H) |
| 1 | 1 | 1 | 1 | 1 |
| 2 | 1 | 2 | 2 | 2 |
| 3 | 1 | 3 | 3 | 3 |
| 4 | 2 | 1 | 2 | 3 |
| 5 | 2 | 2 | 3 | 1 |
| 6 | 2 | 3 | 1 | 2 |
| 7 | 3 | 1 | 3 | 2 |
| 8 | 3 | 2 | 1 | 3 |
| 9 | 3 | 3 | 2 | 1 |

**Table S4** Parameters and levels of the *L*_9_(3^4^) orthogonal experiment method.

| Parameters | Level 1 | Level 2 | Level 3 |
| --- | --- | --- | --- |
| (E) Time (h) | 2.50 | 3.00 | 3.50 |
| (F) Sucrose (g) | 1.700 | 1.750 | 1.800 |
| (G) Salt (g) | 1.875 | 2.000 | 2.125 |
| (H) Mixed spice (g) | 1.450 | 1.500 | 1.550 |
